# Pharmacogenomic landscape in Thailand: Array-based profiling and EMR-linked medication exposure

**DOI:** 10.1371/journal.pone.0355201

**Published:** 2026-08-03

**Authors:** Phongthana Pasookhush, Sophida Suta, Sureeporn Pumeiam, Pichanun Mongkolsucharitkul, Bonggochpass Pinsawas, Suphawan Ophakas, Korapat Mayurasakorn

**Affiliations:** 1 Siriraj Population Health and Nutrition Research Group, Research Department, Faculty of Medicine Siriraj Hospital, Mahidol University, Bangkok, Thailand; 2 Siriraj Center of Research Excellence for Diabetes and Obesity (SiCORE-DO), Faculty of Medicine Siriraj Hospital, Mahidol University, Bangkok, Thailand; INNN: Instituto Nacional de Neurologia y Neurocirugia Manuel Velasco Suarez, MEXICO

## Abstract

Pharmacogenomic (PGx) data in Thailand remain limited, and genetics-only surveys rarely quantify “realized actionability”—the overlap between actionable PGx phenotypes and real-world medication exposure. We profiled 4,662 Thai adults using SNP-array data and a pre-specified PGx panel (11 genes; 26 markers) with a hybrid required/optional calling policy for diplotype/phenotype assignment. CPIC level A/B gene–drug relationships were linked to hospital electronic medical record (EMR) prescription/dispensation data to quantify drug-specific realized actionability. Overall callability across gene-results was 98.62%, exceeding 99% for most genes and lower for *CYP2C19* (95.99%) and *NUDT15* (90.28%). Across nine phenotype-coded genes, 95.99% carried ≥1 CPIC-actionable result (median 2; IQR 2–3). Actionable prevalence among callable individuals was highest for *CYP3A5* (58.54%) and *CYP2C19* (56.67%), followed by *ABCG2* (45.10%) and *UGT1A1* (27.37%). EMR linkage identified 1,529 (32.58%) participants exposed to ≥1 study medication; omeprazole (n = 658) and statins were most common (atorvastatin n = 606; simvastatin n = 603). Among users, actionable phenotypes were frequent for *CYP2C19*–omeprazole (55.02%) and *SLCO1B1*–statins (21.95–23.05%). In conclusion, an Asian-optimized SNP array supports scalable PGx phenotyping in Thai adults. EMR linkage quantifies realized actionability and highlights high-yield targets (*CYP2C19*–proton pump inhibitors; *SLCO1B1*–statins) for pre-emptive implementation.

## Introduction

Inter-individual variability in drug response is a common challenge in clinical practice. Patients treated with the same medication at standard doses may experience different levels of efficacy, toxicity, or both [1,2]. This variability is influenced by many factors, including age, sex, comorbidities, concomitant medications, environmental exposures, and biological determinants such as genetic and epigenetic variation [3]. Pharmacogenomics (PGx) offers a practical approach to precision therapeutics by linking inherited variation in pharmacogenes to predictable differences in drug metabolism, transport, and exposure [4]. In this way, PGx can help guide more individualized drug selection and dosing, with the aim of reducing preventable adverse drug reactions and improving treatment benefit [5]. However, the clinical value of PGx depends not only on genotyping but also on standardized interpretation and prescribing guidance. The Clinical Pharmacogenetics Implementation Consortium (CPIC) publishes peer-reviewed guidelines that translate genotype results into prescribing recommendations (e.g., dose adjustment or alternative therapy) [6,7]. The Pharmacogene Variation (PharmVar) consortium complements this effort by standardizing star-allele nomenclature, which supports consistent genotype-to-phenotype translation and harmonized reporting across laboratories and studies [8,9].

Large-scale biobank studies have demonstrated that clinically actionable PGx variation is common and that many individuals are exposed to medications with established guideline-based recommendations, supporting the potential value of pre-emptive PGx testing at the population level [10]. However, implementation priorities cannot be assumed to be the same across populations. Allele and phenotype distributions vary across ancestries, and extrapolating frequency estimates from predominantly European datasets may be misleading [11]. In addition, differences in disease burden and prescribing patterns influence which gene–drug pairs are most commonly encountered in routine care. As a result, implementation priorities depend on local medication utilization patterns as well as genetics [11]. In Thailand, a whole-genome sequencing (WGS) study has provided an important baseline overview of pharmacogenomic variation [12]. Nonetheless, genetics-only surveys do not quantify “realized actionability”, defined here as the intersection between actionable PGx phenotypes and observed medication exposure within a healthcare system. They also do not directly address how well scalable genotyping approaches can support implementation-facing PGx reporting.

To address these gaps, we performed an array-based PGx analysis in two Thai adult cohorts linked to hospital care and curated a pre-specified panel of clinically relevant pharmacogenes captured on the Infinium Asian Screening Array (ASA), using CPIC/PharmVar-aligned definitions. Our primary objective was to characterize gene-level callability and the distribution of PGx diplotypes and phenotypes in this cohort. Our secondary objective was to link CPIC level A/B gene–drug relationships to routinely collected electronic medical record (EMR) medication data to quantify realized actionability in routine care. By integrating population PGx frequencies with medication exposure in a real-world hospital setting, this study aims to inform implementation priorities by identifying high-frequency gene–drug pairs with high expected clinical reach for pre-emptive screening and guideline-driven prescribing workflows in Thailand.

## Materials and methods

### Study cohort and genotyping

We conducted a secondary analysis of two prospective studies: the Siriraj Health (SIH) [13] study and the Siriraj OneHealth (SIOH) study [14]. Both studies enrolled adults employed at Siriraj Hospital or residing in surrounding urban communities with the aim of investigating non-communicable diseases. Medication records were retrieved from the hospital EMR system as part of routine care and scheduled health checkups. All procedures adhered to the Declaration of Helsinki and were approved by the Institutional Review Board of the Faculty of Medicine Siriraj Hospital, Mahidol University (current study COA no. Si 235/2026). Cohort approvals included COA no. Si 647/2016 for the SIH cohort, and COA no. Si 381/2023 and Si 631/2019 for the SIOH cohort. Written informed consent was obtained from all participants at enrollment in the original studies. Genotype data from the original cohort datasets were accessed for research purposes on 4 June 2024. EMR data for this secondary analysis were accessed for research purposes on 1 April 2026, following Institutional Review Board approval of the current study.

Genotyping was performed using the Infinium Asian Screening Array (ASA) v1.0 (Illumina, USA). Genotype quality control (QC) followed our previous work with minor modifications [14,15] and was conducted using PLINK v1.9 [16]. Participants with sex discrepancies were excluded, duplicated variants were removed, variants with call rate <90% were excluded, and participants with call rate <97% were removed. Kinship was estimated using the kinship-based inference for genome-wide association studies (KING) robust algorithm [17], and one individual from each pair of second-degree relatives or closer (kinship coefficient >0.086) was excluded to minimize bias in population frequency estimates. Hardy–Weinberg equilibrium filtering was not applied to PGx panel variants to avoid removing phenotype-defining markers. Variant coordinates were left-aligned and normalized to GRCh38 using bcftools v1.20 [18]. No genotype imputation was performed. The QC-passed, GRCh38-aligned dataset was used for downstream pharmacogenomic calling.

### Pharmacogenomic panel

We constructed a pre-specified pharmacogenomic panel comprising 11 clinically relevant genes covered by the ASA v1.0: *CYP2C19* [19], *CYP2C9* [20–22]*, CYP3A5* [23]*, SLCO1B1* [20], *ABCG2* [20]*, VKORC1* [22], *CYP4F2* [22]*, TPMT* [24]*, NUDT15* [24]*, UGT1A1* [25] and CYP2B6 [26] (S1 Table). Genes were selected based on the presence of CPIC-referenced markers on the array and the availability of CPIC guidelines with evidence level A or B for at least one medication used in the hospital setting. Genes requiring complex structural resolution (e.g., *CYP2D6*) were not included in the primary array-based panel.

For each gene, we curated a minimal set of array markers from the ASA v1.0 manifest and mapped rsIDs to star-allele and functional definitions using CPIC and PharmVar resources. Markers were categorized as “required” or “optional” for star-allele determination. Required markers were necessary to distinguish CPIC-relevant star alleles and support diplotype assignment, whereas optional markers refined allele subtypes without changing CPIC phenotype category (S1 Table).

### Diplotype and phenotype calling

Diplotype and phenotype calling were performed on the post-QC, GRCh38-aligned genotype dataset using the pharmacogenomic panel definitions above. For each gene, genotypes at panel markers were extracted from the VCF and interpreted in a reference/alternate-aware manner. Within each gene, genotype patterns at required markers (and optional markers when available) were matched to a gene-specific star-allele definition table (S1 Table).

External statistical phasing was not performed. Allele definitions were implemented in a phase-insensitive manner when cis/trans configuration did not alter functional interpretation under CPIC phenotype categories. For TPMT, only the common decreased-function alleles *2, *3B and *3C were considered; genotype patterns were mapped to TPMT metabolizer status without distinguishing configurations such as *3A (cis combination of *3B and *3C) [24]. For *UGT1A1*, rs887829 (*80) was used as a tag for the *28 haplotype [25,27]. For *CYP2B6*, the *6 allele was approximated using the available markers rs3745274 and rs2279343 [26] (S1and S2 Tables). These proxy definitions may not capture rarer alleles not represented on the array.

A diplotype was assigned for each gene when a unique compatible combination could be identified. Diplotypes were translated into clinical phenotypes using gene-specific diplotype-to-phenotype mapping rules aligned to CPIC categories (S2 Tables). At the gene level, we applied a hybrid required/optional calling policy: a gene was considered called when all required markers were genotyped and consistent with panel definitions; if required markers were present but one or more optional markers were missing, a phenotype was assigned and flagged. If any required marker was missing or the genotype pattern did not match defined allele combinations, the gene was labeled undetermined and no phenotype was assigned. Callability (called/limited/undetermined) was summarized for each gene and used to define N_called (called + limited) denominators.

### Electronic medical record data and CPIC actionability

EMR-derived medication exposures were ascertained from 1 January 2014–31 December 2024. Medication records were extracted for a pre-defined list of study drugs across all available strengths and formulations prescribed and/or dispensed by the hospital. A user was defined as having ≥1 prescription and/or dispensation record for a given drug during the observation period. Drug names were normalized to generic ingredients, and fixed-dose combinations were decomposed into constituent ingredients while retaining original product identifiers for traceability (S3 Table). For primary analyses, repeated records for the same individual and medication were collapsed into a participant-level ever-use indicator (0/1) for each medication.

### Statistical analysis

All analyses were descriptive. For each pharmacogene, phenotype frequencies were calculated among callable participants (N_called) and reported as proportions with Wilson 95% confidence intervals. Gene-level callability (called, limited, undetermined) was summarized using counts and percentages. Participant-level burden was summarized as the number of CPIC-actionable genes per person and reported using counts and percentages, together with the median and interquartile range.

For EMR-linked analyses, medication exposure was summarized as the number of users per medication (≥1 prescription/dispensation record from 2014–2024). Realized pharmacogenomic actionability was quantified for each CPIC level A/B drug–gene pair as the proportion of users carrying an actionable phenotype for the mapped gene (N_actionable_users/N_users). Individuals could contribute to multiple drug–gene pairs if they received multiple medications; drug-specific proportions were calculated independently within each medication’s user group and were not aggregated across drugs. For warfarin, *VKORC1* rs9923231 and *CYP4F2* rs2108622 genotype distributions and *CYP2C9* phenotypes were summarized descriptively among warfarin users due to small sample size. Analyses were conducted in R v4.4.2. Visualizations were generated using ggplot2 and finalized in Adobe Illustrator v29.1 (Adobe, USA)

## Results

### Study cohort and pharmacogenomic panel callability

**Fig 1:**
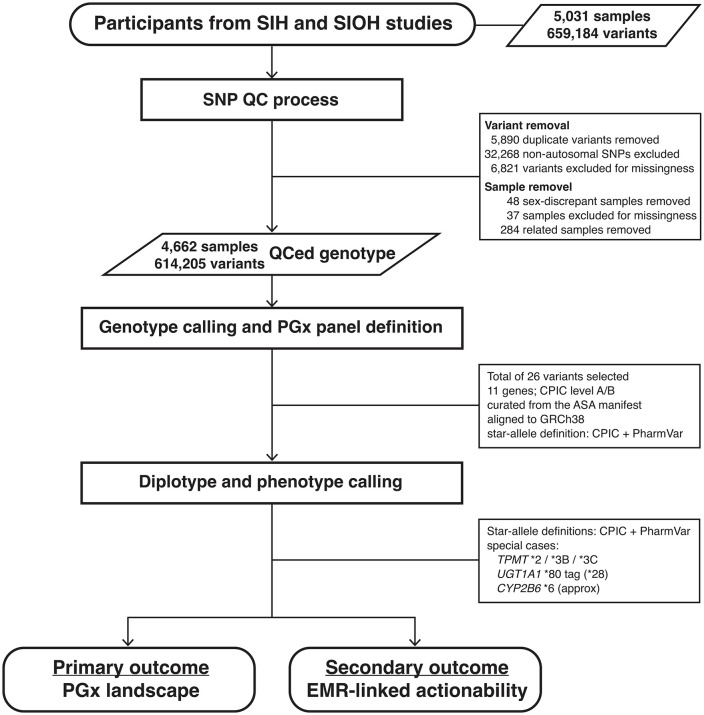
Study workflow for array-based pharmacogenomic calling and EMR linkage. Individuals from the Siriraj Health (SIH) and Siriraj OneHealth (SIOH) studies were genotyped using the Infinium Asian Screening Array (ASA) v1.0 (Illumina, USA). After genotype quality control (removal of duplicate variants, sex-discrepant samples, high-missingness variants/samples, and related individuals), 4,662 individuals with QC-passed genotypes were retained. A pre-specified pharmacogenomic panel (11 genes; 26 variants) was curated from CPIC level A/B gene-drug relationships and PharmVar/CPIC star-allele definitions and aligned to GRCh38. Diplotypes and phenotypes were assigned using a hybrid required/optional marker policy with pre-defined handling of selected special cases (*TPMT* *2/*3B/*3C; *UGT1A1* *80 tag; *CYP2B6* *6 approximation). The primary outcome was the population pharmacogenomic landscape, and the secondary outcome was EMR-linked pharmacogenomic actionability.

A total of 5,031 participants from the SIH and SIOH studies with ASA genotyping data were available. After genotype QC (removal of duplicate variants, sex-discrepant participants, high-missingness variants and participants, and related individuals), 4,662 participants and 614,205 variants remained for analysis. A pre-specified pharmacogenomic panel comprising 26 variants across 11 genes was extracted from the QC-passed genotype dataset for downstream diplotype and phenotype calling (Fig 1).

Using a hybrid required/optional marker policy, gene-level callability was high across most genes, with an overall callability of 98.62% across all gene-by-participant results (Table 1). Call rates (N_called/4,662) were 100% for *CYP4F2*, *CYP3A5*, and *UGT1A1*, and ≥99% for *VKORC1, CYP2C9, SLCO1B1, ABCG2, TPMT*, and *CYP2B6*. Lower callability was observed for *CYP2C19* (N_called = 4,475; 95.99%) and *NUDT15* (N_called = 4,209; 90.28%), reflecting a higher proportion of undetermined results at phenotype-defining markers. Marker-level allele frequencies and missingness for each panel variant are provided in Supplementary S6 Table. Overall, the high callability of the 26 panel variants supports the feasibility of using the ASA array for population-scale pharmacogenomic phenotype assignment in this cohort.

**Table 1:** Pharmacogenomic panel callability and denominators for phenotype frequency analyses in the cohort (n = 4,662).

Gene	Called	Limited	Undetermined	N_called	Call rate (%)
*VKORC1*	4,659	0	3	4,659	99.94
*CYP4F2*	4,662	0	0	4,662	100.00
*CYP2C9*	4,641	14	7	4,655	99.85
*CYP2C19*	4,428	47	187	4,475	95.99
*CYP3A5*	4,662	0	0	4,662	100.00
*SLCO1B1*	4,660	0	2	4,660	99.96
*ABCG2*	4,661	0	1	4,661	99.98
*TPMT*	4,647	0	15	4,647	99.68
*NUDT15*	4,203	6	453	4,209	90.28
*UGT1A1*	4,662	0	0	4,662	100.00
*CYP2B6*	4,622	0	40	4,622	99.14
Sum across gene-results	50,507	67	708	50,574	98.62

Called: all required markers present and matched a defined allele pattern.

Limited: required markers present; ≥ 1 optional marker missing (phenotype assigned).

Undetermined: ≥ 1 required marker missing.

N_called = called + limited.

Call rate (%) = 100 x N_called/4,662.

Overall call rate (%) = 100 x (∑ N_called across genes)/ (11 x 4,662).

Counts in the final row represent the sum of per-gene called across 11 genes.

### Distribution of pharmacogenomic phenotypes and warfarin-related loci

**Fig 2:**
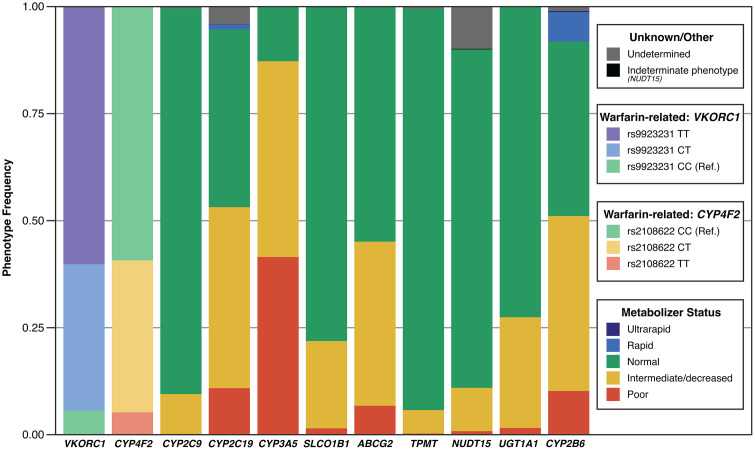
Distribution of pharmacogenomic phenotypes and key warfarin-related genotypes in the study cohort (n = 4,662). Stacked bars show the proportion of individuals in each metabolizer/function category for *CYP2C19, CYP2C9, CYP3A5, SLCO1B1, ABCG2, TPMT, NUDT15, UGT1A1*, and *CYP2B6*, calculated among individuals with an assigned call for that gene (N_called; called + limited). Two warfarin-related loci, *VKORC1* (rs9923231) and *CYP4F2* (rs2108622), are shown as genotype categories. “Undetermined” denotes that no diplotype could be assigned due to missing required markers or unmatched patterns, whereas “Indeterminate phenotype (*NUDT15*)” indicates that a diplotype was assigned but mapped to an indeterminate *NUDT15* phenotype under the pre-specified rules. Colors reflect clinical directionality where applicable (reduced function/sensitivity in yellow/red, normal in green, increased function/sensitivity in blue/purple, and unknown/other in greyscale).

Gene-level phenotype distributions were summarized among callable participants for each gene (N_called) using the pre-specified calling and mapping rules (Fig 2 and Supplementary S4 Table); corresponding diplotype frequencies are reported in Supplementary S5 Table. Normal function/metabolizer phenotypes represented the largest proportion of callable results across genes, with varying contributions from decreased/intermediate and poor categories. For *CYP2C19* (N_called = 4,475), intermediate and normal metabolizer phenotypes were similarly frequent (44.2% and 43.3%, respectively), while 11.2% were poor metabolizers; rapid and ultrarapid metabolizers were uncommon (1.2% and 0.1%). *CYP2C9* results were predominantly normal metabolizer (90.6%; N_called = 4,655), with 9.4% intermediate and 0.02% poor metabolizers. For *CYP3A5* (N_called = 4,662), intermediate (45.9%) and poor metabolizer phenotypes (41.5%) were more common than normal metabolizers (12.6%) (Fig 2 and Supplementary S4 Table). Together, these distributions indicate that clinically relevant non-normal metabolizer phenotypes—particularly for *CYP2C19* and *CYP3A5*—are common in the cohort.

For transporter genes, *SLCO1B1* (N_called = 4,660) showed 20.5% decreased function and 1.3% poor function, while *ABCG2* (N_called = 4,661) showed 38.5% decreased function and 6.6% poor function. *TPMT* was largely normal (94.4%; N_called = 4,647), with 5.5% intermediate metabolizers and 0.1% classified as intermediate-or-poor metabolizer. For *NUDT15* (N_called = 4,209), 11.3% were intermediate metabolizers and 0.7% poor metabolizers; an additional 0.2% were assigned an indeterminate phenotype despite an assigned diplotype (*1/*4) under the pre-specified mapping rules. *UGT1A1* (N_called = 4,662) demonstrated 25.9% intermediate and 1.4% poor metabolizer phenotypes. For *CYP2B6* (N_called = 4,622), intermediate and normal metabolizers were similarly frequent (41.4% and 41.2%), with 10.2% poor, 7.0% rapid, and 0.3% ultrarapid metabolizers (Fig 2 and Supplementary Table S4). These gene-specific patterns suggest substantial potential for CPIC-guided prescribing across multiple therapeutic areas, motivating subsequent analyses of actionability burden and EMR-linked drug exposure.

Warfarin-related loci were summarized as genotype distributions rather than CPIC metabolizer phenotypes. For *VKORC1* rs9923231 (N_called = 4,659), the cohort comprised TT 60.2%, CT 34.4%, and CC 5.5%, where the rs9923231 T allele is associated with increased warfarin sensitivity. For *CYP4F2* rs2108622 (N_called = 4,662), genotypes were CC 59.3%, CT 35.6%, and TT 5.1%, where the rs2108622 T allele is associated with decreased warfarin sensitivity (Fig. 2 and Supplementary S4 Table). These common warfarin-related genotypes provide a genetic basis for subsequent EMR-linked evaluation among anticoagulant users.

### Burden and prevalence of CPIC-actionable pharmacogenomic phenotypes

**Fig 3:**
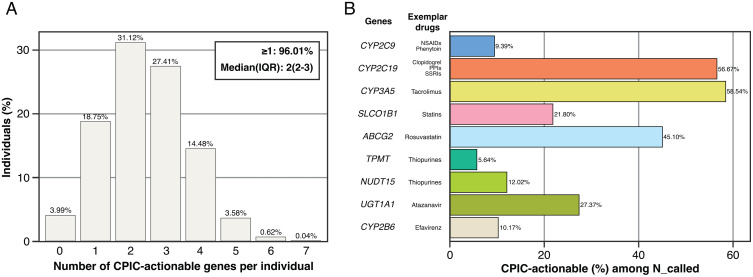
Burden and per-gene prevalence of CPIC-actionable pharmacogenomic phenotypes in the cohort. Distribution of the number of CPIC-actionable genes per individual, reported as percentages (A). Actionability was assessed using pre-specified CPIC level A/B gene-drug relationships and phenotype-based actionability rules. Individuals contributed to a given gene only if the gene was callable; phenotypes labeled Undetermined and *NUDT15* indeterminate phenotypes were excluded from actionability classification. Percentage of participants with a CPIC-actionable phenotype for each gene, calculated among N_called (B). Genes included: C*YP2C9, CYP2C19, CYP3A5, SLCO1B1, ABCG2, TPMT, NUDT15, UGT1A1*, and *CYP2B6*. Exemplar CPIC-guided medications are shown for clinical context. Warfarin-related loci (*VKORC1* and *CYP4F2*) were not included in this figure and were evaluated in drug-exposure analyses.

We quantified the burden of CPIC-actionable pharmacogenomic results across nine phenotype-coded genes (excluding *VKORC1* and *CYP4F2*) using pre-specified CPIC level A/B gene–drug relationships and phenotype-based actionability rules (Fig 3). Actionability was summarized at the participant level (number of actionable genes per participant) and the gene level (proportion actionable among callable participants, N_called).

At the participant level, 96.01% of participants had at least one CPIC-actionable result. The distribution was centered at 2–3 actionable genes per participant: 31.12% had two actionable genes and 27.41% had three; 14.48% and 3.58% had four and five actionable genes, respectively. Only 3.99% had no actionable results across the nine genes (Fig 3A and Supplementary S7 Table).

At the gene level, the prevalence of actionable phenotypes varied substantially (Fig 3B and Supplementary S7 Table). The highest proportions were observed for *CYP3A5* (58.54% actionable among N_called) and *CYP2C19* (56.67%), followed by *ABCG2* (45.10%) and *UGT1A1* (27.37%). Actionable phenotypes were also present for *SLCO1B1* (21.80%) and *NUDT15* (12.02%), while lower actionable proportions were observed for *CYP2B6* (10.17%), *CYP2C9* (9.39%), and *TPMT* (5.64%). These results indicate that a large proportion of the cohort carries actionable genotypes in genes linked to commonly used medication classes, providing a basis for subsequent EMR-linked analyses. Warfarin-related loci (*VKORC1* and *CYP4F2*) were not included in this actionability analysis and are evaluated in the drug-exposure analyses.

### Medication exposure and realized pharmacogenomic actionability (EMR-linked)

**Fig 4:**
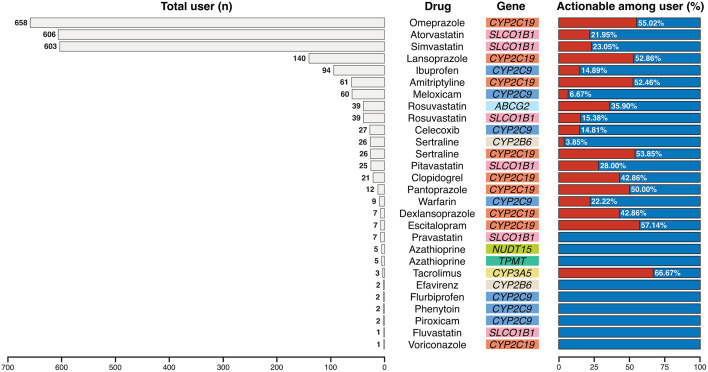
Medication exposure and realized pharmacogenomic actionability in the cohort (2014-2024). The left panel shows the total number of participants with ≥1 prescription and/or dispensation record for each medication during the observation period (“Total users”). For each drug–gene pair, the right panel shows the proportion of users carrying an actionable pharmacogenomic phenotype for the mapped gene (“Actionable among users”), where actionable phenotypes were defined using CPIC level A/B gene–drug relationships and the study’s phenotype-based actionability rules. Colored gene labels indicate the pharmacogene contributing to each drug row. Some medications appear more than once when multiple genes are relevant (e.g., rosuvastatin and sertraline). Participants could contribute to multiple rows if they received multiple medications; percentages are calculated independently within each drug’s user group.

Medication exposure was ascertained from EMR records during 2014–2024 for the pre-specified CPIC level A/B drug list in the full genotyped cohort (n = 4,662). Overall, 1,529 (32.58%) participants had at least one prescription and/or dispensation record for a study medication. The most commonly prescribed/dispensed medications were omeprazole (n = 658) and statins, including atorvastatin (n = 606) and simvastatin (n = 603) (Fig 4). Denominators for drug–gene actionability analyses reflect users with a callable phenotype for the mapped gene.

Among users of each medication, the proportion carrying an actionable phenotype for the mapped gene varied by drug–gene pair. Actionable *CYP2C19* phenotypes were frequent among users of proton pump inhibitors (PPIs) and other *CYP2C19*-guided therapies, including omeprazole (362/658, 55.02%) and lansoprazole (74/140, 52.86%), as well as amitriptyline (32/61, 52.46%) and clopidogrel (9/21, 42.86%). For statins, actionable SLCO1B1 phenotypes were observed in atorvastatin users (133/606, 21.95%) and simvastatin users (139/602, 23.05%). Among rosuvastatin users (n = 39), actionable phenotypes were observed for *ABCG2* (14/39, 35.90%) and *SLCO1B1* (6/39, 15.38%), reflecting the multi-gene relevance of rosuvastatin. For *CYP2C9*-guided NSAIDs, actionable phenotypes were observed in a smaller fraction of users, including ibuprofen (14/94, 14.89%) and celecoxib (4/27, 14.81%).

Warfarin exposure was uncommon (n = 9). Based on *CYP2C9* phenotype alone, 2/9 (22.22%) users carried a CPIC-actionable *CYP2C9* phenotype. However, all warfarin users carried at least one *VKORC1* rs9923231 T allele (CT/TT), and 3/9 carried *CYP4F2* rs2108622 CT, indicating that genetic factors incorporated in CPIC warfarin dosing guidance were common among exposed participants despite the small number of users (Supplementary S8 Table).

## Discussion

This study provides an implementation-oriented PGx profile of Thai adults by combining array-based PGx calling with EMR linkage. Using a CPIC/PharmVar-aligned, pre-specified panel (11 genes; 26 markers) assayed on the Infinium Asian Screening Array, we observed high gene-level callability across the cohort, supporting the feasibility of scalable SNP-array genotyping for population-scale PGx profiling in this setting. We then characterized phenotype distributions and found that clinically relevant non-normal phenotypes were common—particularly for *CYP2C19* and *CYP3A5*—and that most participants carried at least one CPIC-actionable PGx result across nine phenotype-coded genes. Extending beyond genetics-only summaries, we quantified “realized actionability” by intersecting actionable phenotypes with real-world medication exposure in hospital care: proton pump inhibitors and statins were the most commonly prescribed/dispensed medication classes among the study medications, and actionable phenotypes were frequently observed among users for high-priority gene–drug pairs (e.g., *CYP2C19*–PPIs and *SLCO1B1*–statins). Together, these findings move from population allele/phenotype frequencies toward implementation readiness by highlighting high-yield gene–drug combinations that could be prioritized for pre-emptive testing and guideline-driven prescribing pathways in Thailand.

SNP-array genotyping offers a pragmatic route for scaling PGx implementation because it can deliver high callability for selected, clinically actionable markers at substantially lower cost than WGS [28,29]. In this cohort, the high call rates across the pre-specified panel support the feasibility of using an Asian-optimized array to generate implementation-ready PGx results at population scale, with transparent per-gene denominators that make missingness and uncertainty explicit. This favorable cost-to-coverage profile is particularly relevant in resource-constrained settings, where the ability to return actionable results for high-priority gene–drug pairs may outweigh the incremental value of broader variant discovery. However, array-based PGx necessarily trades breadth for scalability [30]. The panel does not capture genes requiring complex structural resolution (e.g., *CYP2D6*) and cannot resolve all star-allele subtypes or rare functional variants [31]. In addition, selected definitions relied on proxy markers (e.g., UGT1A1*80 as a tag) and phase-insensitive rules for combinations that do not materially alter phenotype classification [24–27]. These limitations should be considered when translating results to clinical reporting and underscore the need for targeted confirmatory testing or expanded assays for specific clinical indications, even as array-based approaches enable low-cost deployment of pre-emptive PGx in routine care [30].

Our population PGx profile aligns with previous Thai studies but differs from predominantly European datasets, underscoring the need for population-specific frequency estimates [10]. A recent WGS analysis of 949 unrelated Thai individuals provided a comprehensive baseline of pharmacogenomic variation and reported that clinically significant/actionable diplotypes are common across multiple clinically relevant genes, including *CYP2C19*, *CYP3A5*, *SLCO1B1*, and *VKORC1* [12]. A Thai-focused *CYP2C19* study emphasized the diversity of *CYP2C19* polymorphisms in Thailand and differences relative to other populations, aligning with our finding that intermediate and poor metabolizer phenotypes are frequent and clinically relevant in this setting [32]. More recent Thai and regional reports have also emphasized the importance of population-specific PGx resources and local implementation frameworks for translating pharmacogenomic evidence into clinical practice [33]. Across overlapping pharmacogenes, these comparisons suggest that a targeted SNP-array panel can capture major population patterns needed for implementation-facing PGx reporting [34]. At the same time, WGS interrogates a broader spectrum of rare variants and complex loci that are not fully represented on arrays, and “actionability” definitions can differ between studies [12]. Consistent with this, *TPMT* showed lower frequencies of clinically significant diplotypes in the WGS survey and similarly low actionable proportions in our cohort, whereas several high-yield genes (e.g., *CYP2C19*, *CYP3A5*, *SLCO1B1*) remain prominent across both approaches [12]. Finally, large-scale biobank analyses such as the UK Biobank—comprising predominantly European ancestry participants—have demonstrated substantially different allele and phenotype distributions across pharmacogenes [10]. More recent population-based PGx studies have similarly shown that the prevalence of actionable variation and the prioritization of gene–drug pairs can vary meaningfully across ancestries and healthcare settings [35]. Together, these findings reinforce that Thai-specific frequency estimates, alongside local medication utilization patterns, are needed to prioritize gene–drug pairs for implementation and to avoid misleading extrapolation from overrepresented reference populations.

A key contribution of this work is the quantification of “realized actionability,” which links actionable PGx phenotypes to observed medication exposure in routine hospital care [10]. In the EMR-linked analysis, proton pump inhibitors and statins accounted for the largest numbers of exposed participants, highlighting these drug classes as high-yield targets for PGx-enabled prescribing pathways in this setting. Recent large-cohort studies have also shown that clinically relevant PGx variants can be linked to real-world prescribing patterns and used to prioritize drug–gene pairs for implementation at scale [35]. Notably, statin exposure in our cohort was substantial and consistent with routine-care expectations for cardiometabolic risk management [36,37], supporting that the EMR-derived medication data capture clinically meaningful prescribing patterns rather than rare exposures. Among omeprazole users, more than half carried an actionable *CYP2C19* phenotype, consistent with the high prevalence of non-normal *CYP2C19* metabolizer status in Thai populations and underscoring the potential impact of PGx-guided acid-suppressive therapy [32]. Similarly, approximately one-fifth to one-quarter of statin users carried actionable *SLCO1B1* phenotypes, and over one-third of rosuvastatin users carried actionable *ABCG2* phenotypes, supporting the relevance of transporter-mediated PGx considerations for lipid-lowering therapy in routine practice [20]. These findings are also consistent with more recent real-world studies supporting the continued clinical relevance of transporter-related PGx variation in statin prescribing and statin-related adverse effects [38]. By presenting actionable proportions among users together with absolute exposure counts, our results provide an empiric framework for prioritizing gene–drug pairs for pre-emptive testing based on expected clinical reach (number of users) and potential PGx impact (actionable fraction), rather than genetics alone.

This study has several strengths that support its implementation focus. First, we combined array-based PGx calling with EMR linkage, allowing us to move beyond population frequency reporting and quantify realized actionability in routine care. Second, the PGx panel and actionability rules were pre-specified and aligned to CPIC/PharmVar resources, improving interpretability and reproducibility for implementation planning. Third, high gene-level callability across the panel—reported with transparent per-gene denominators—supports the feasibility of scalable PGx reporting from an Asian-optimized SNP array in this setting. Several limitations should also be considered. First, PGx inference was limited to variants represented on the ASA panel; thus, some pharmacogenes and rare functional alleles were not evaluated, and certain star-allele assignments relied on proxy markers as described above. EMR medication ascertainment reflects prescriptions/dispensations captured within a single hospital system and an ever-use definition; exposure may be under-ascertained for over-the-counter medications (particularly NSAIDs such as ibuprofen) and does not capture adherence or longitudinal dose adjustments [39,40]. In contrast, under-ascertainment of proton pump inhibitor exposure may be less pronounced because patients with persistent gastroesophageal reflux symptoms commonly seek medical evaluation and receive prescriptions within the hospital system [41,42]. Finally, the EMR-linked analyses quantify genotype–exposure overlap rather than clinical effectiveness or adverse drug reaction outcomes, and estimates for less commonly prescribed medications (including warfarin) were limited by small user counts. Together, these considerations suggest that the results are best interpreted as implementation signals that can inform prioritization of high-yield gene–drug pairs and motivate outcome-linked and multi-site evaluations.

In summary, this study shows that a targeted, Asian-optimized SNP-array panel can generate high-callability PGx phenotypes in Thai adults and that CPIC-actionable variation is common in genes linked to widely used therapies. By linking actionable phenotypes to EMR-derived medication exposure, we provide an implementation-relevant estimate of where PGx could most frequently intersect with routine prescribing in a Thai hospital setting—particularly for *CYP2C19*-guided proton pump inhibitor use and transporter-related statin therapy (*SLCO1B1* and *ABCG2*). These results can support near-term prioritization of gene–drug pairs for pre-emptive testing and decision support. Future work should expand coverage to complex pharmacogenes not captured by arrays (e.g., *CYP2D6*), improve medication capture across care settings (including over-the-counter use), and evaluate outcome- and dose-linked endpoints to quantify clinical benefit and cost-effectiveness in real-world practice.

## Supporting information

S1 Table Pharmacogenomic marker manifest and calling roles (required/optional) for the 11-gene ASA-based panel (GRCh38). (PDF)

S2 Table Diplotype/genotype-to-phenotype mapping rules applied for PGx phenotype assignment in the 11-gene panel. (PDF)

S3 Table CPIC level A/B drug–gene relationships used for EMR-linked realized actionability analyses. (PDF)

S4 Table Pharmacogenomic phenotype and warfarin-related genotype category frequencies in the cohort (N_total= 4,662), with Wilson 95% confidence intervals. (PDF)

S5 Table Observed diplotype and genotype-category frequencies in the cohort (N_total = 4,662), with Wilson 95% confidence intervals. (PDF)

S6 Table Allele frequencies of pharmacogenomic panel markers in the cohort (N_total = 4,662), with Wilson 95% confidence intervals. (PDF)

S7 Table Participant-level burden and gene-level prevalence of CPIC-actionable pharmacogenomic phenotypes in the cohort (N_total = 4,662). (PDF)

S8 Table Warfarin-related pharmacogenomic profiles among warfarin users (n = 9). (PDF)
