# Supplementary material for: Pharmacogenomic landscape in Thailand: Array-based profiling and EMR-linked medication exposure
Source: PLoS One. 2026 Aug 3;21(8):e0355201. doi: 10.1371/journal.pone.0355201 (PMC13432136; doi:10.1371/journal.pone.0355201)
Supplement: S1 Table — (PDF) [file pone.0355201.s001.pdf]

Supplementary Table S1. Pharmacogenomic marker manifest and calling roles (required/optional) for the 11-gene ASA-based panel (GRCh38).

Gene	Marker/ Star allele	rsID	Position (GRCh38)	Required/ optional	Functional annotation
CYP2C19	*2	rs4244285	10:94781859	Required	Decreased function
CYP2C19	*3	rs4986893	10:94780653	Required	Decreased function
CYP2C19	*4	rs28399504	10:94762706	Optional	Decreased function
CYP2C19	*5	rs56337013	10:94852738	Optional	Decreased function
CYP2C19	*6	rs72552267	10:94775453	Optional	Decreased function
CYP2C19	*8	rs41291556	10:94775416	Optional	Decreased function
CYP2C19	*17	rs12248560	10:94761900	Required	Increased function
CYP2C9	*2	rs1799853	10:94942290	Required	Decreased function
CYP2C9	*3	rs1057910	10:94981296	Required	Decreased function
CYP2C9	*5	rs28371686	10:94981301	Optional	Decreased function
CYP2C9	*8	rs7900194	10:94942309	Optional	Decreased function
CYP2C9	*11	rs28371685	10:94981224	Optional	Decreased function
CYP3A5	*3	rs776746	7:99672916	Required	Decreased function
CYP3A5	*6	rs10264272	7:99665212	Optional	Decreased function
SLCO1B1	*5/*15	rs4149056	12:21178615	Required	Decreased function
ABCG2	rs2231142	rs2231142	4:88131171	Required	Decreased function
VKORC1	rs9923231	rs9923231	16:31096368	Required	Warfarin increased sensitivity (T)
CYP4F2	*3	rs2108622	19:15879621	Required	Warfarin decreased sensitivity
NUDT15	*3	rs116855232	13:48045719	Required	Decreased function
NUDT15	*4	rs147390019	13:48045720	Optional	Decreased function
TPMT[†]	*2	rs1800462	6:18143724	Required	Decreased function
TPMT[†]	*3B	rs1800460	6:18138997	Required	Decreased function
TPMT[†]	*3C	rs1142345	6:18130687	Required	Decreased function
UGT1A1[‡]	*80	rs887829	2:233759924	Required	Decreased function
CYP2B6[§]	*6	rs2279343	19:41009358	Required	Component of *6 approximation
CYP2B6[§]	*6	rs3745274	19:41006936	Required	Component of *6 approximation

[†] *TPMT*: *3A not resolved (phase); *3B and *3C treated as decreased-function alleles for phenotype assignment
[‡] *UGT1A1*: rs887829 (*80) used as a tag for *28
[§] *CYP2B6*: *6 approximated using rs3745274 and rs2279343
