# Supplementary material for: Pharmacogenomic landscape in Thailand: Array-based profiling and EMR-linked medication exposure
Source: PLoS One. 2026 Aug 3;21(8):e0355201. doi: 10.1371/journal.pone.0355201 (PMC13432136; doi:10.1371/journal.pone.0355201)
Supplement: S2 Table — (PDF) [file pone.0355201.s002.pdf]

Supplementary Table S2. Diplotype/genotype-to-phenotype mapping rules applied for PGx phenotype assignment in the 11-gene panel

Gene	Diplotype/genotype pattern	Coded diplotype/phenotype summary
CYP2C19	*1/*1	CYP2C19 Normal Metabolizer
CYP2C19	*1/*2	CYP2C19 Intermediate Metabolizer
CYP2C19	*1/*3	CYP2C19 Intermediate Metabolizer
CYP2C19	*1/*4	CYP2C19 Intermediate Metabolizer
CYP2C19	*1/*5	CYP2C19 Intermediate Metabolizer
CYP2C19	*1/*6	CYP2C19 Intermediate Metabolizer
CYP2C19	*1/*8	CYP2C19 Intermediate Metabolizer
CYP2C19	*1/*17	CYP2C19 Rapid Metabolizer
CYP2C19	*2/*2	CYP2C19 Poor Metabolizer
CYP2C19	*2/*3	CYP2C19 Poor Metabolizer
CYP2C19	*2/*4	CYP2C19 Poor Metabolizer
CYP2C19	*2/*5	CYP2C19 Poor Metabolizer
CYP2C19	*2/*6	CYP2C19 Poor Metabolizer
CYP2C19	*2/*8	CYP2C19 Poor Metabolizer
CYP2C19	*2/*17	CYP2C19 Intermediate Metabolizer
CYP2C19	*3/*3	CYP2C19 Poor Metabolizer
CYP2C19	*3/*4	CYP2C19 Poor Metabolizer
CYP2C19	*3/*5	CYP2C19 Poor Metabolizer
CYP2C19	*3/*6	CYP2C19 Poor Metabolizer
CYP2C19	*3/*8	CYP2C19 Poor Metabolizer
CYP2C19	*3/*17	CYP2C19 Intermediate Metabolizer
CYP2C19	*4/*4	CYP2C19 Poor Metabolizer
CYP2C19	*4/*5	CYP2C19 Poor Metabolizer
CYP2C19	*4/*6	CYP2C19 Poor Metabolizer
CYP2C19	*4/*8	CYP2C19 Poor Metabolizer
CYP2C19	*4/*17	CYP2C19 Intermediate Metabolizer
CYP2C19	*5/*5	CYP2C19 Poor Metabolizer
CYP2C19	*5/*6	CYP2C19 Poor Metabolizer
CYP2C19	*5/*8	CYP2C19 Poor Metabolizer
CYP2C19	*5/*17	CYP2C19 Intermediate Metabolizer
CYP2C19	*6/*6	CYP2C19 Poor Metabolizer
CYP2C19	*6/*8	CYP2C19 Poor Metabolizer
CYP2C19	*6/*17	CYP2C19 Intermediate Metabolizer
CYP2C19	*8/*8	CYP2C19 Poor Metabolizer
CYP2C19	*8/*17	CYP2C19 Intermediate Metabolizer
CYP2C19	*17/*17	CYP2C19 Ultrarapid Metabolizer

CYP2C9	*1/*1	CYP2C9 Normal Metabolizer
CYP2C9	*1/*2	CYP2C9 Intermediate Metabolizer
CYP2C9	*1/*3	CYP2C9 Intermediate Metabolizer
CYP2C9	*1/*5	CYP2C9 Intermediate Metabolizer
CYP2C9	*1/*8	CYP2C9 Intermediate Metabolizer
CYP2C9	*1/*11	CYP2C9 Intermediate Metabolizer
CYP2C9	*2/*2	CYP2C9 Intermediate Metabolizer
CYP2C9	*2/*3	CYP2C9 Poor Metabolizer
CYP2C9	*2/*5	CYP2C9 Intermediate Metabolizer
CYP2C9	*2/*8	CYP2C9 Intermediate Metabolizer
CYP2C9	*2/*11	CYP2C9 Intermediate Metabolizer
CYP2C9	*3/*3	CYP2C9 Poor Metabolizer
CYP2C9	*3/*5	CYP2C9 Poor Metabolizer
CYP2C9	*3/*8	CYP2C9 Poor Metabolizer
CYP2C9	*3/*11	CYP2C9 Poor Metabolizer
CYP2C9	*5/*5	CYP2C9 Intermediate Metabolizer
CYP2C9	*5/*8	CYP2C9 Intermediate Metabolizer
CYP2C9	*8/*8	CYP2C9 Intermediate Metabolizer
CYP2C9	*8/*11	CYP2C9 Intermediate Metabolizer
CYP2C9	*11/*11	CYP2C9 Intermediate Metabolizer
CYP3A5	*1/*1	CYP3A5 Normal Metabolizer
CYP3A5	*1/*3	CYP3A5 Intermediate Metabolizer
CYP3A5	*1/*6	CYP3A5 Intermediate Metabolizer
CYP3A5	*3/*3	CYP3A5 Poor Metabolizer
CYP3A5	*3/*6	CYP3A5 Poor Metabolizer
CYP3A5	*6/*6	CYP3A5 Poor Metabolizer
SLCO1B1	*1/*1	SLCO1B1 Normal Function
SLCO1B1	*1/*5	SLCO1B1 Decreased Function
SLCO1B1	*1/*15	SLCO1B1 Decreased Function
SLCO1B1	*5/*5	SLCO1B1 Poor Function
SLCO1B1	*5/*15	SLCO1B1 Poor Function
SLCO1B1	*15/*15	SLCO1B1 Poor Function
ABCG2	rs2231142 reference (G)/rs2231142 reference (G)	ABCG2 Normal Function
ABCG2	rs2231142 reference (G)/rs2231142 variant (T)	ABCG2 Decreased Function
ABCG2	rs2231142 variant (T)/rs2231142 variant (T)	ABCG2 Poor Function
VKORC1	rs9923231 reference (C)/rs9923231 reference (C)	Warfarin normal sensitivity
VKORC1	rs9923231 reference (C)/rs9923231 variant (T)	Warfarin increased sensitivity
VKORC1	rs9923231 variant (T)/rs9923231 variant (T)	Warfarin increased sensitivity *2
CYP4F2	rs2108622 reference (C)/rs2108622 reference (C)	Warfarin normal sensitivity

CYP4F2	rs2108622 reference (C)/rs2108622 variant (T)	Warfarin decreased sensitivity
CYP4F2	rs2108622 variant (T)/rs2108622 variant (T)	Warfarin decreased sensitivity *2
NUDT15	*1/*1	NUDT15 Normal Metabolizer
NUDT15	*1/*3	NUDT15 Intermediate Metabolizer
NUDT15	*1/*4	NUDT15 Indeterminate
NUDT15	*3/*3	NUDT15 Poor Metabolizer
NUDT15	*3/*4	NUDT15 Possible Intermediate Metabolizer
NUDT15	*4/*4	NUDT15 Indeterminate
TPMT†	*1/*1	TPMT Normal Metabolizer
TPMT†	*1/*2	TPMT Intermediate Metabolizer
TPMT†	*1/*3A	TPMT Intermediate Metabolizer
TPMT†	*1/*3B	TPMT Intermediate Metabolizer
TPMT†	*1/*3C	TPMT Intermediate Metabolizer
TPMT†	*2/*2	TPMT Poor Metabolizer
TPMT†	*2/*3A	TPMT Poor Metabolizer
TPMT†	*2/*3B	TPMT Poor Metabolizer
TPMT†	*2/*3C	TPMT Poor Metabolizer
TPMT†	*3A/*3A	TPMT Poor Metabolizer
TPMT†	*3A/*3B	TPMT Poor Metabolizer
TPMT†	*3A/*3C	TPMT Poor Metabolizer
UGT1A1‡	*1/*1	UGT1A1 Normal Metabolizer
UGT1A1‡	*1/*80	UGT1A1 Indeterminate
UGT1A1‡	*1/*80+*28	UGT1A1 Intermediate Metabolizer
UGT1A1‡	*80/*80	UGT1A1 Indeterminate
UGT1A1‡	*80/*80+*28	UGT1A1 Indeterminate
UGT1A1‡	*80+*28/*80+*28	UGT1A1 Poor Metabolizer
CYP2B6§	*1/*1	CYP2B6 Normal Metabolizer
CYP2B6§	*4/*4	CYP2B6 Ultrarapid Metabolizer
CYP2B6§	*1/*4	CYP2B6 Rapid Metabolizer
CYP2B6§	*1/*6	CYP2B6 Intermediate Metabolizer
CYP2B6§	*1/*9	CYP2B6 Intermediate Metabolizer
CYP2B6§	*4/*6	CYP2B6 Intermediate Metabolizer
CYP2B6§	*4/*9	CYP2B6 Intermediate Metabolizer
CYP2B6§	*6/*6	CYP2B6 Poor Metabolizer
CYP2B6§	*6/*9	CYP2B6 Poor Metabolizer

†*TPMT*: *3A was not explicitly resolved due to phase ambiguity; diplotypes involving *3B (rs1800460) and *3C (rs1142345) were treated as decreased-function alleles for phenotype assignment

‡*UGT1A1*: rs887829 (*80) was used as a tag for the *28 haplotype; diplotypes containing "*80+*28" represent the tagged decreased-function haplotype under this array-based definition.

§*CYP2B6*: The *6 allele was approximated using rs3745274 and rs2279343 without external phasing. For double-heterozygous genotypes where phase could not be determined, compatible diplotypes were reported as a phase-ambiguous category (e.g., "*1/*6 or *4/*9 (phase unknown)") according to the study rule set
