# Supplementary material for: Pharmacogenomic landscape in Thailand: Array-based profiling and EMR-linked medication exposure
Source: PLoS One. 2026 Aug 3;21(8):e0355201. doi: 10.1371/journal.pone.0355201 (PMC13432136; doi:10.1371/journal.pone.0355201)
Supplement: S3 Table — (PDF) [file pone.0355201.s003.pdf]

Supplementary Table S3. CPIC level A/B drug–gene relationships used for EMR-linked realized actionability analyses.

Gene	Drug	CPIC level	CPIC publications (PMID)	CPIC guideline (URL)
CYP2C19	amitriptyline	A	23486447;27997040	https://cpicpgx.org/guidelines/guideline-for-tricyclic-antidepressants-and-cyp2d6-and-cyp2c19/
CYP2C19	citalopram	A	25974703;37032427	https://cpicpgx.org/guidelines/cpic-guideline-for-ssri-and-snri-antidepressants/
CYP2C19	clopidogrel	A	21716271;23698643;35034351	https://cpicpgx.org/guidelines/guideline-for-clopidogrel-and-cyp2c19/
CYP2C19	escitalopram	A	25974703;37032427	https://cpicpgx.org/guidelines/cpic-guideline-for-ssri-and-snri-antidepressants/
CYP2C19	lansoprazole	A	32770672	https://cpicpgx.org/guidelines/cpic-guideline-for-proton-pump-inhibitors-and-cyp2c19/
CYP2C19	omeprazole	A	32770672	https://cpicpgx.org/guidelines/cpic-guideline-for-proton-pump-inhibitors-and-cyp2c19/
CYP2C19	pantoprazole	A	32770672	https://cpicpgx.org/guidelines/cpic-guideline-for-proton-pump-inhibitors-and-cyp2c19/
CYP2C19	sertraline	A	25974703;37032427	https://cpicpgx.org/guidelines/cpic-guideline-for-ssri-and-snri-antidepressants/
CYP2C19	voriconazole	A	27981572	https://cpicpgx.org/guidelines/guideline-for-voriconazole-and-cyp2c19/
CYP2C9	celecoxib	A	32189324	https://cpicpgx.org/guidelines/cpic-guideline-for-nsaids-based-on-cyp2c9-genotype/
CYP2C9	flurbiprofen	A	32189324	https://cpicpgx.org/guidelines/cpic-guideline-for-nsaids-based-on-cyp2c9-genotype/
CYP2C9	fluvastatin	A	35152405	https://cpicpgx.org/guidelines/cpic-guideline-for-statins/
CYP2C9	fosphenytoin	A	25099164;32779747	https://cpicpgx.org/guidelines/guideline-for-phenytoin-and-cyp2c9-and-hla-b/
CYP2C9	ibuprofen	A	32189324	https://cpicpgx.org/guidelines/cpic-guideline-for-nsaids-based-on-cyp2c9-genotype/
CYP2C9	lornoxicam	A	32189324	https://cpicpgx.org/guidelines/cpic-guideline-for-nsaids-based-on-cyp2c9-genotype/
CYP2C9	meloxicam	A	32189324	https://cpicpgx.org/guidelines/cpic-guideline-for-nsaids-based-on-cyp2c9-genotype/
CYP2C9	phenytoin	A	25099164;32779747	https://cpicpgx.org/guidelines/guideline-for-phenytoin-and-cyp2c9-and-hla-b/
CYP2C9	piroxicam	A	32189324	https://cpicpgx.org/guidelines/cpic-guideline-for-nsaids-based-on-cyp2c9-genotype/
CYP2C9	siponimod	A	Not available	Not available
CYP2C9	tenoxicam	A	32189324	https://cpicpgx.org/guidelines/cpic-guideline-for-nsaids-based-on-cyp2c9-genotype/
CYP2C9	warfarin	A	21900891;28198005	https://cpicpgx.org/guidelines/guideline-for-warfarin-and-cyp2c9-and-vkorc1/

CYP3A5	tacrolimus	A	25801146	https://cpicpgx.org/guidelines/guideline-for-tacrolimus-and-cyp3a5/
SLCO1B1	atorvastatin	A	35152405	https://cpicpgx.org/guidelines/cpic-guideline-for-statins/
SLCO1B1	fluvastatin	A	35152405	https://cpicpgx.org/guidelines/cpic-guideline-for-statins/
SLCO1B1	lovastatin	A	35152405	https://cpicpgx.org/guidelines/cpic-guideline-for-statins/
SLCO1B1	pitavastatin	A	35152405	https://cpicpgx.org/guidelines/cpic-guideline-for-statins/
SLCO1B1	pravastatin	A	35152405	https://cpicpgx.org/guidelines/cpic-guideline-for-statins/
SLCO1B1	rosuvastatin	A	35152405	https://cpicpgx.org/guidelines/cpic-guideline-for-statins/
SLCO1B1	simvastatin	A	22617227;24918167;35152405	https://cpicpgx.org/guidelines/cpic-guideline-for-statins/
ABCG2	rosuvastatin	A	35152405	https://cpicpgx.org/guidelines/cpic-guideline-for-statins/
VKORC1	warfarin	A	21900891;28198005	https://cpicpgx.org/guidelines/guideline-for-warfarin-and-cyp2c9-and-vkorc1/
CYP4F2	warfarin	A	21900891;28198005	https://cpicpgx.org/guidelines/guideline-for-warfarin-and-cyp2c9-and-vkorc1/
NUDT15	azathioprine	A	21270794;23422873;30447069	https://cpicpgx.org/guidelines/guideline-for-thiopurines-and-tpmt/
NUDT15	mercaptopurine	A	21270794;23422873;30447069	https://cpicpgx.org/guidelines/guideline-for-thiopurines-and-tpmt/
NUDT15	thioguanine	A	21270794;23422873;30447069	https://cpicpgx.org/guidelines/guideline-for-thiopurines-and-tpmt/
TPMT	azathioprine	A	21270794;23422873;30447069	https://cpicpgx.org/guidelines/guideline-for-thiopurines-and-tpmt/
TPMT	mercaptopurine	A	21270794;23422873;30447069	https://cpicpgx.org/guidelines/guideline-for-thiopurines-and-tpmt/
TPMT	thioguanine	A	21270794;23422873;30447069	https://cpicpgx.org/guidelines/guideline-for-thiopurines-and-tpmt/
UGT1A1	atazanavir	A	26417955	https://cpicpgx.org/guidelines/guideline-for-atazanavir-and-ugt1a1/
UGT1A1	irinotecan	A	Not available	Not available
CYP2B6	efavirenz	A	31006110	https://cpicpgx.org/guidelines/cpic-guideline-for-efavirenz-based-on-cyp2b6-genotype/

Only CPIC level A/B gene–drug pairs were included in the EMR-linked realized actionability analyses

Some drugs appear more than once because multiple genes are relevant (e.g., rosuvastatin with *SLCO1B1* and *ABCG2*; warfarin with *CYP2C9*, *VKORC1*, and *CYP4F2*)
