# Supplementary material for: Pharmacogenomic landscape in Thailand: Array-based profiling and EMR-linked medication exposure
Source: PLoS One. 2026 Aug 3;21(8):e0355201. doi: 10.1371/journal.pone.0355201 (PMC13432136; doi:10.1371/journal.pone.0355201)
Supplement: S4 Table — (PDF) [file pone.0355201.s004.pdf]

Supplementary Table S4. Pharmacogenomic phenotype and warfarin-related genotype category frequencies in the cohort (N_total = 4,662), with Wilson 95% confidence intervals.

Gene	Phenotype	Count	N_called[†]	Frequency (N_called)	Wilson 95% CI	Frequency (N_total)
CYP2C19	CYP2C19 Ultrarapid Metabolizer	3	4,475	0.001	0–0.002	0.001
CYP2C19	CYP2C19 Rapid Metabolizer	54	4,475	0.012	0.009–0.016	0.012
CYP2C19	CYP2C19 Normal Metabolizer	1,939	4,475	0.433	0.419–0.448	0.416
CYP2C19	CYP2C19 Intermediate Metabolizer	1,978	4,475	0.442	0.428–0.457	0.424
CYP2C19	CYP2C19 Poor Metabolizer	501	4,475	0.112	0.103–0.122	0.107
CYP2C19	Undetermined[‡]	187	4,475			0.004
CYP2C9	CYP2C9 Normal Metabolizer	4,218	4,655	0.906	0.897–0.914	0.905
CYP2C9	CYP2C9 Intermediate Metabolizer	436	4,655	0.094	0.086–0.102	0.094
CYP2C9	CYP2C9 Poor Metabolizer	1	4,655	0	0–0.001	0.000
CYP2C9	Undetermined[‡]	7	4,655			0.002
CYP3A5	CYP3A5 Normal Metabolizer	589	4,662	0.126	0.117–0.136	0.126
CYP3A5	CYP3A5 Intermediate Metabolizer	2,140	4,662	0.459	0.445–0.473	0.459
CYP3A5	CYP3A5 Poor Metabolizer	1,933	4,662	0.415	0.401–0.429	0.415
SLCO1B1	SLCO1B1 Normal Function	3,644	4,660	0.782	0.770–0.794	0.782
SLCO1B1	SLCO1B1 Decreased Function	954	4,660	0.205	0.193–0.217	0.205
SLCO1B1	SLCO1B1 Poor Function	62	4,660	0.013	0.010–0.017	0.013
SLCO1B1	Undetermined[‡]	2	4,660			0.000
ABCG2	ABCG2 Normal Function	2,559	4,661	0.549	0.535–0.563	0.549
ABCG2	ABCG2 Decreased Function	1,793	4,661	0.385	0.371–0.399	0.385
ABCG2	ABCG2 Poor Function	309	4,661	0.066	0.060–0.074	0.066
ABCG2	Undetermined[‡]	1	4,661			0.000
VKORC1	warfarin normal sensitivity	255	4,659	0.055	0.049–0.062	0.055
VKORC1	warfarin increased sensitivity	1,601	4,659	0.344	0.330–0.357	0.343
VKORC1	warfarin increased sensitivity *2	2,803	4,659	0.602	0.587–0.616	0.601
VKORC1	Undetermined[‡]	3	4,659			0.001
CYP4F2	warfarin normal sensitivity	2,765	4,662	0.593	0.579–0.607	0.593
CYP4F2	warfarin decreased sensitivity	1,660	4,662	0.356	0.342–0.370	0.356
CYP4F2	warfarin decreased sensitivity *2	237	4,662	0.051	0.045–0.058	0.051
NUDT15	NUDT15 Normal Metabolizer	3,697	4,209	0.878	0.868–0.888	0.793
NUDT15	NUDT15 Intermediate Metabolizer	474	4,209	0.113	0.103–0.123	0.102
NUDT15	NUDT15 Poor Metabolizer	31	4,209	0.007	0.005–0.010	0.007
NUDT15	NUDT15 Indeterminate	7	4,209	0.002	0.001–0.003	0.002
NUDT15	Undetermined[‡]	453	4,209			0.097
TPMT	TPMT Normal Metabolizer	4,385	4,647	0.944	0.937–0.905	0.941
TPMT	TPMT Intermediate Metabolizer	257	4,647	0.055	0.049–0.062	0.055

TPMT	TPMT Intermediate or Poor Metabolizer	5	4,647	0.001	0–0.003	0.001
TPMT	Undetermined‡	15	4,647			0.003
UGT1A1	UGT1A1 Normal Metabolizer	3,386	4,662	0.726	0.713–0.739	0.726
UGT1A1	UGT1A1 Intermediate Metabolizer	1,209	4,662	0.259	0.247–0.272	0.259
UGT1A1	UGT1A1 Poor Metabolizer	67	4,662	0.014	0.011–0.018	0.014
CYP2B6	CYP2B6 Ultrarapid Metabolizer	13	4,622	0.003	0.002–0.005	0.003
CYP2B6	CYP2B6 Rapid Metabolizer	322	4,622	0.070	0.063–0.077	0.069
CYP2B6	CYP2B6 Normal Metabolizer	1,905	4,622	0.412	0.398–0.426	0.409
CYP2B6	CYP2B6 Intermediate Metabolizer	1,912	4,622	0.414	0.400–0.428	0.410
CYP2B6	CYP2B6 Poor Metabolizer	470	4,622	0.102	0.093–0.111	0.101
CYP2B6	Undetermined‡	40	4,622			0.009

†N_called denotes callable participants for that gene (called + limited).
‡Undetermined indicates no phenotype/genotype category could be assigned due to missing required markers or unmatched patterns; "Undetermined" rows are reported as a proportion of N_total only.
