# Supplementary material for: Pharmacogenomic landscape in Thailand: Array-based profiling and EMR-linked medication exposure
Source: PLoS One. 2026 Aug 3;21(8):e0355201. doi: 10.1371/journal.pone.0355201 (PMC13432136; doi:10.1371/journal.pone.0355201)
Supplement: S5 Table — (PDF) [file pone.0355201.s005.pdf]

Supplementary Table S5. Observed diplotype and genotype-category frequencies in the cohort (N_total = 4,662), with Wilson 95% confidence intervals.

Gene	Diplotype	Count	N_called[†]	Frequency (N_called)	Wilson 95% CI	Frequency (N_total)
CYP2C19	*1/*1	1,939	4,475	0.433	0.419–0.448	0.416
CYP2C19	*1/*2	1,722	4,475	0.385	0.371–0.399	0.369
CYP2C19	*1/*3	247	4,475	0.055	0.049–0.062	0.053
CYP2C19	*1/*5	8	4,475	0.002	0.001–0.004	0.002
CYP2C19	*1/*8	1	4,475	0.000	0.000–0.001	0.000
CYP2C19	*1/*17	54	4,475	0.012	0.009–0.016	0.012
CYP2C19	*2/*2	366	4,475	0.082	0.074–0.090	0.079
CYP2C19	*2/*3	114	4,475	0.025	0.021–0.031	0.024
CYP2C19	*2/*5	8	4,475	0.002	0.001–0.004	0.002
CYP2C19	*2/*6	2	4,475	0.000	0.000–0.002	0.000
CYP2C19	*2/*8	1	4,475	0.000	0.000–0.001	0.000
CYP2C19	*3/*3	10	4,475	0.002	0.001–0.004	0.002
CYP2C19	*17/*17	3	4,475	0.001	0.000–0.002	0.001
CYP2C19	Undetermined[‡]	187	4,475			0.040
CYP2C9	*1/*1	4,218	4,655	0.906	0.897–0.914	0.905
CYP2C9	*1/*2	36	4,655	0.008	0.006–0.011	0.008
CYP2C9	*1/*3	374	4,655	0.080	0.073–0.089	0.080
CYP2C9	*1/*8	25	4,655	0.005	0.004–0.008	0.005
CYP2C9	*1/*11	1	4,655	0.000	0.000–0.001	0.000
CYP2C9	*3/*8	1	4,655	0.000	0.000–0.001	0.000
CYP2C9	Undetermined[‡]	7	4,655			0.002
CYP3A5	*1/*1	589	4,662	0.126	0.117–0.136	0.126
CYP3A5	*1/*3	2,140	4,662	0.459	0.445–0.473	0.459
CYP3A5	*3/*3	1,933	4,662	0.415	0.401–0.429	0.415
SLCO1B1	*1/*1	3,644	4,660	0.782	0.770–0.794	0.782
SLCO1B1	*1/*5 or *1/*15	954	4,660	0.205	0.193–0.217	0.205
SLCO1B1	*5/*5 or *15/*15	62	4,660	0.013	0.010–0.017	0.013
SLCO1B1	Undetermined[‡]	2	4,660			0.000
ABCG2	rs2231142 reference (C)/ rs2231142 reference (C)	2,559	4,661	0.549	0.535–0.563	0.549
ABCG2	rs2231142 reference (C)/ rs2231142 variant (A)	1,793	4,661	0.385	0.371–0.399	0.385
ABCG2	rs2231142 variant (A)/ rs2231142 variant (A)	309	4,661	0.066	0.060–0.074	0.066
ABCG2	Undetermined[‡]	1	4,661			0.000
VKORC1	rs9923231 reference (C)/ rs9923231 reference (C)	255	4,659	0.055	0.049–0.062	0.055

VKORC1	rs9923231 reference (C)/ rs9923231 variant (T)	1,601	4,659	0.344	0.330–0.357	0.343
VKORC1	rs9923231 variant (T)/ rs9923231 variant (T)	2,803	4,659	0.602	0.587–0.616	0.601
VKORC1	Undetermined‡	3	4,659			0.001
CYP4F2	rs2108622 reference (G)/ rs2108622 reference (G)	2,765	4,662	0.593	0.579–0.607	0.593
CYP4F2	rs2108622 reference (G)/ rs2108622 variant (A)	1,660	4,662	0.356	0.342–0.370	0.356
CYP4F2	rs2108622 variant (A)/ rs2108622 variant (A)	237	4,662	0.051	0.045–0.058	0.051
NUDT15	*1/*1	3,697	4,209	0.878	0.868–0.888	0.793
NUDT15	*1/*3	474	4,209	0.113	0.103–0.123	0.102
NUDT15	*1/*4	7	4,209	0.002	0.001–0.003	0.002
NUDT15	*3/*3	31	4,209	0.007	0.005–0.010	0.007
NUDT15	Undetermined‡	453	4,209			0.097
TPMT	*1/*1	4,385	4,647	0.944	0.937–0.950	0.941
TPMT	*1/*3A (phase unknown)	5	4,647	0.001	0.000–0.003	0.001
TPMT	*1/*3C	257	4,647	0.055	0.049–0.062	0.055
TPMT	Undetermined‡	15	4,647			0.003
UGT1A1	*1/*1	3,386	4,662	0.726	0.713–0.739	0.726
UGT1A1	*1/*80+*28	1,209	4,662	0.259	0.247–0.272	0.259
UGT1A1	*80+*28/*80+*28	67	4,662	0.014	0.011–0.018	0.014
CYP2B6	*1/*1	1,905	4,622	0.412	0.398–0.426	0.409
CYP2B6	*1/*4	322	4,622	0.070	0.063–0.077	0.069
CYP2B6	*1/*6 or *4/*9 (phase unknown)	1,771	4,622	0.383	0.369–0.397	0.380
CYP2B6	*1/*9	11	4,622	0.002	0.001–0.004	0.002
CYP2B6	*4/*4	13	4,622	0.003	0.002–0.005	0.003
CYP2B6	*6/*4 (phase certain)	130	4,622	0.028	0.024–0.033	0.028
CYP2B6	*6/*6	469	4,622	0.101	0.093–0.111	0.101
CYP2B6	*6/*9 (phase certain)	1	4,622	0.000	0.000–0.001	0.000
CYP2B6	Undetermined‡	40	4,622			0.009

†N_called denotes callable participants for that gene (called + limited).

‡Undetermined indicates no phenotype/genotype category could be assigned due to missing required markers or unmatched patterns; “Undetermined” rows are reported as a proportion of N_total only.
