# Supplementary material for: Pharmacogenomic landscape in Thailand: Array-based profiling and EMR-linked medication exposure
Source: PLoS One. 2026 Aug 3;21(8):e0355201. doi: 10.1371/journal.pone.0355201 (PMC13432136; doi:10.1371/journal.pone.0355201)
Supplement: S6 Table — (PDF) [file pone.0355201.s006.pdf]

Supplementary Table S6. Allele frequencies of pharmacogenomic panel markers in the cohort (N_total = 4,662), with Wilson 95% confidence intervals.

Gene	Marker/ Star allele	N_genotyped	N_missing	ALT allele (n)	ALT allele frequency	Wilson 95% CI
CYP2C19	*2	4,606	56	2626	0.285	0.276–0.294
CYP2C19	*3	4,661	1	386	0.041	0.038–0.046
CYP2C19	*4	4,658	4	0	0.000	0.000–0.000
CYP2C19	*5	4,662	0	17	0.002	0.001–0.003
CYP2C19	*6	4,617	45	5	0.001	0.000–0.001
CYP2C19	*8	4,662	0	2	0.000	0.000–0.001
CYP2C19	*17	4,531	131	83	0.009	0.007–0.011
CYP2C9	*2	4,662	0	36	0.004	0.003–0.005
CYP2C9	*3	4,655	7	375	0.040	0.036–0.044
CYP2C9	*5	4,655	7	0	0.000	0.000–0.000
CYP2C9	*8	4,659	3	26	0.003	0.002–0.004
CYP2C9	*11	4,658	4	1	0.000	0.000–0.001
CYP3A5	*3	4,662	0	6006	0.644	0.634–0.654
CYP3A5	*6	4,662	0	0	0.000	0.000–0.000
SLCO1B1	*5/*15	4,660	2	1078	0.116	0.109–0.122
ABCG2	rs2231142	4,661	1	2411	0.259	0.250–0.268
VKORC1	rs9923231	4,659	3	7207	0.773	0.765–0.782
CYP4F2	*3	4,662	0	2134	0.229	0.220–0.238
NUDT15	*3	4,209	453	536	0.064	0.059–0.069
NUDT15	*4	4,653	9	7	0.001	0.000–0.002
TPMT[†]	*2	4,661	1	0	0.000	0.000–0.000
TPMT[†]	*3B	4,662	0	5	0.001	0.000–0.001
TPMT[†]	*3C	4,648	14	262	0.028	0.025–0.032
UGT1A1[‡]	*80	4,662	0	1343	0.144	0.137–0.151
CYP2B6[§]	*6	4,657	5	3348	0.359	0.350–0.369
CYP2B6[§]	*6	4,626	36	2854	0.308	0.299–0.318

The rsIDs, GRCh38 positions, and REF/ALT alleles for each marker are reported in Supplementary Table S1. ALT allele frequency was calculated as ALT alleles / (2 × N_genotyped). Wilson 95% confidence intervals were calculated for ALT allele frequency.
