# Supplementary material for: Pharmacogenomic landscape in Thailand: Array-based profiling and EMR-linked medication exposure
Source: PLoS One. 2026 Aug 3;21(8):e0355201. doi: 10.1371/journal.pone.0355201 (PMC13432136; doi:10.1371/journal.pone.0355201)
Supplement: S7 Table — (PDF) [file pone.0355201.s007.pdf]

Supplementary Table S7. Participant-level burden and gene-level prevalence of CPIC-actionable pharmacogenomic phenotypes in the cohort (N_total = 4,662).

Participant-level burden of actionable genes (nine phenotype-coded genes*)

Actionable genes per participant	N_individuals	% of cohort
0	186	3.99
1	874	18.75
2	1,451	31.12
3	1,278	27.41
4	675	14.48
5	167	3.58
6	29	0.62
7	2	0.04
**Total**	**4662**	**100.00**

Gene-level CPIC actionability among callable participants (N_called)

Gene	N_called†	N_actionable	% actionable among N_called
CYP2C9	4,655	437	9.39
CYP2C19	4,475	2,536	56.67
CYP3A5	4,662	2,729	58.54
SLCO1B1	4,660	1,016	21.80
ABCG2	4,661	2,102	45.10
TPMT	4,647	262	5.64
NUDT15	4,202	505	12.02
UGT1A1	4,662	1,276	27.37
CYP2B6	4,622	470	10.17

*Nine phenotype-coded genes: *CYP2C19, CYP2C9, CYP3A5, SLCO1B1, ABCG2, TPMT, NUDT15, UGT1A1, CYP2B6. VKORC1* and *CYP4F2* were excluded because they were summarized as genotype categories rather than CPIC metabolizer phenotypes.

†N_called denotes callable participants for that gene (called + limited). For actionability classification, participants with Undetermined phenotypes (and *NUDT15* Indeterminate) were excluded from the numerator/denominator.
