# Supplementary material for: Pharmacogenomic landscape in Thailand: Array-based profiling and EMR-linked medication exposure
Source: PLoS One. 2026 Aug 3;21(8):e0355201. doi: 10.1371/journal.pone.0355201 (PMC13432136; doi:10.1371/journal.pone.0355201)
Supplement: S8 Table — (PDF) [file pone.0355201.s008.pdf]

Supplementary Table S8. Warfarin-related pharmacogenomic profiles among warfarin users (n = 9).

Case	CYP2C9 phenotype	VKORC1 rs9923231 (C/T)	CYP4F2 rs2108622 (C/T)
1	CYP2C9 Normal Metabolizer	CT	CC
2	CYP2C9 Normal Metabolizer	TT	CT
3	CYP2C9 Normal Metabolizer	TT	CT
4	CYP2C9 Normal Metabolizer	CT	CC
5	CYP2C9 Normal Metabolizer	CT	CC
6	CYP2C9 Intermediate Metabolizer	TT	CC
7	CYP2C9 Normal Metabolizer	TT	CT
8	CYP2C9 Normal Metabolizer	CT	CC
9	CYP2C9 Intermediate Metabolizer	TT	CC

Case numbers are arbitrary labels created for presentation only and do not correspond to participant identifiers or any study record number.
